# Immunogenicity and safety of one-dose human papillomavirus vaccine compared with two or three doses in Tanzanian girls (DoRIS): an open-label, randomised, non-inferiority trial

**DOI:** 10.1016/S2214-109X(22)00309-6

**Published:** 2022-09-13

**Authors:** Deborah Watson-Jones, John Changalucha, Hilary Whitworth, Ligia Pinto, Paul Mutani, Jackton Indangasi, Troy Kemp, Ramadhan Hashim, Beatrice Kamala, Rebecca Wiggins, Twaib Songoro, Nicholas Connor, Gladys Mbwanji, Miquel A Pavon, Brett Lowe, Devis Mmbando, Saidi Kapiga, Philippe Mayaud, Silvia de SanJosé, Joakim Dillner, Richard J Hayes, Charles J Lacey, Kathy Baisley

**Affiliations:** a Mwanza Intervention Trials Unit, National Institute for Medical Research, Mwanza, Tanzania; b Faculty of Infectious and Tropical Diseases, London School of Hygiene & Tropical Medicine, London, UK; c Faculty of Epidemiology and Population Health, London School of Hygiene & Tropical Medicine, London, UK; d HPV Serology Laboratory, Frederick National Laboratory for Cancer Research, Leidos Biomedical Research, Frederick, MD, USA; e York Biomedical Research Institute & Hull York Medical School, University of York, York, UK; f Infection and Cancer Laboratory, Cancer Epidemiology Research Programme, ICO-IDIBELL, L’Hospitalet de Llobregat, Barcelona, Spain; g Centro de Investigación Biomédica en Red de Epidemiología y Salud Pública, Madrid, Spain; h Unit of Infections and Cancer Cancer Epidemiology Research Programme, Institut Català d’ Oncologia, Barcelona, Spain; i National Cancer Institute, Rockville, USA; j Karolinska Institute, Stockholm, Sweden

## Abstract

**Background:**

An estimated 15% of girls aged 9–14 years worldwide have been vaccinated against human papillomavirus (HPV) with the recommended two-dose or three-dose schedules. A one-dose HPV vaccine schedule would be simpler and cheaper to deliver. We report immunogenicity and safety results of different doses of two different HPV vaccines in Tanzanian girls.

**Methods:**

In this open-label, randomised, phase 3, non-inferiority trial, we enrolled healthy schoolgirls aged 9–14 years from Government schools in Mwanza, Tanzania. Eligible participants were randomly assigned to receive one, two, or three doses of either the 2-valent vaccine (Cervarix, GSK Biologicals, Rixensart) or the 9-valent vaccine (Gardasil-9, Sanofi Pasteur MSD, Lyon). The primary outcome was HPV 16 specific or HPV 18 specific seropositivity following one dose compared with two or three doses of the same HPV vaccine 24 months after vaccination. Safety was assessed as solicited adverse events up to 30 days after each dose and unsolicited adverse events up to 24 months after vaccination or to last study visit. The primary outcome was done in the per-protocol population, and safety was analysed in the total vaccinated population. This study was registered in ClinicalTrials.gov, NCT02834637.

**Findings:**

Between Feb 23, 2017, and Jan 6, 2018, we screened 1002 girls for eligibility. 72 girls were excluded. 930 girls were enrolled and randomly assigned to receive one dose of Cervarix (155 participants), two doses of Cervarix (155 participants), three doses of Cervarix (155 participants), one dose of Gardasil-9 (155 participants), two doses of Gardasil-9 (155 participants), or three doses of Gardasil-9 (155 participants). 922 participants received all scheduled doses within the defined window (three withdrew, one was lost to follow-up, and one died before completion; two received their 6-month doses early, and one received the wrong valent vaccine in error; all 930 participants were included in the total vaccinated cohort). Retention at 24 months was 918 (99%) of 930 participants. In the according-to-protocol cohort, at 24 months, 99% of participants who received one dose of either HPV vaccine were seropositive for HPV 16 IgG antibodies, compared with 100% of participants who received two doses, and 100% of participants who received three doses. This met the prespecified non-inferiority criteria. Anti-HPV 18 seropositivity at 24 months did not meet non-inferiority criteria for one dose compared to two doses or three doses for either vaccine, although more than 98% of girls in all groups had HPV 18 antibodies. 53 serious adverse events (SAEs) were experienced by 42 (4·5%) of 930 girls, the most common of which was hospital admission for malaria. One girl died of malaria. Number of events was similar between groups and no SAEs were considered related to vaccination.

**Interpretation:**

A single dose of the 2-valent or 9-valent HPV vaccine in girls aged 9–14 years induced robust immune responses up to 24 months, suggesting that this reduced dose regimen could be suitable for prevention of HPV infection among girls in the target age group for vaccination.

**Funding:**

UK Department for International Development/UK Medical Research Council/Wellcome Trust Joint Global Health Trials Scheme, The Bill & Melinda Gates Foundation, and the US National Cancer Institute.

**Translation:**

For the KiSwahili translation of the abstract see Supplementary Materials section.

## Introduction

Cervical cancer results in more than 340 000 potentially preventable deaths annually, with most fatalities in low-income and middle-income countries.^1^ Four vaccines are licensed for the prevention of human papillomavirus (HPV), the main cause of cervical cancer. WHO cervical cancer elimination targets include 90% of girls younger than 15 years receiving a prophylactic HPV vaccine by 2030.^2^ In countries that have introduced HPV vaccination, the vaccines are delivered as a multidose schedule with two doses offered to girls younger than 15 years, three doses offered to girls 15 years or older and to immunocompromised individuals, and boys being offered the vaccine in some countries. Barriers to the introduction and uptake of HPV vaccination are greatest in countries that bear the highest burden of cervical cancer morbidity and mortality, particularly the cost of delivering a multidose vaccine schedule.^3^ Only 15% of girls in the target age group for HPV vaccine (9–14 years) worldwide are estimated to be fully vaccinated with the currently recommended two-dose or three-dose schedules.^4^ As with other primary health-care services, HPV vaccine delivery has been disrupted by the COVID-19 pandemic and, in some WHO regions, last dose coverage is less than 5%.^5^

**Research in context**

**Evidence before this study**

Several authors of this work participated in a review to collate the evidence on single dose human papillomavirus (HPV) vaccination. This review identified the absence of evidence from randomised trials and highlighted that data from Africa were also limited. A 2019 systematic review published as part of this evidence review examined the effectiveness and immunogenicity of single dose HPV vaccination among participants who received their HPV vaccine through a clinical trial. Apart from one small randomised trial examining memory B-cell responses following single dose HPV vaccination, results came from observational studies nested within three large HPV vaccine trials (Costa Rica Vaccine Trial [CVT], PATRICIA, and IARC India trial) in which participants did not complete their allocated two-dose or three-dose schedules which resulted in single dose default groups followed up for immunogenicity and efficacy against HPV infection. We did an updated search of MEDLINE, Embase, Global Health Database, and Cochrane Central Register of Controlled Trials from Aug 1, 2018, to Dec 10, 2021, using the search terms “human papillomavirus AND vaccines AND (immunogenicity OR efficacy OR effectiveness) AND dosage”. From this search we identified two additional observational studies that extended the data from two of these studies and, in 2022, results were published from a randomised trial on the efficacy of single dose HPV vaccination in sexually active Kenyan women aged 15–20 years (KEN SHE study). The observational studies showed that frequency of HPV 16 and 18 incident and 12-month persistent infection and vaccine efficacy against infection endpoints was similar in women and girls who received a single dose of vaccine compared with those who received two or three doses. HPV 16 and 18 IgG antibody seropositivity was very high in all dose groups for vaccinated participants, although antibody mean concentrations were lower with one dose than with two or three doses, but remained stable over 11 and 9 years for all doses for two HPV vaccines. HPV infection endpoints were significantly lower in participants who received one vaccine dose compared to unvaccinated controls. The KEN SHE trial showed very high and non-inferior vaccine efficacy for one dose of the 2-valent and 9-valent vaccines compared with a control vaccine at 18 months after the first dose. Non-trials data include an observational cohort study of Ugandan girls who did not complete the 3-dose schedule of the 2-valent vaccine in a Government-administered HPV vaccination demonstration programme. Seroconversion was high for all doses. HPV 16 and 18 binding antibody responses were lower in girls who had received one compared with two or three doses but geometric mean concentrations for one dose recipients were not lower in these Ugandan girls compared with adult women who received one dose in the CVT and in whom efficacy had been demonstrated.

**Added value of this study**

This study is the first randomised clinical trial examining immune responses and safety of single dose HPV vaccine with either the 2-valent or 9-valent vaccine compared with two and three doses of the same vaccines in girls in the target age group of 9–14 years for vaccination. Antibody responses were comparable with those seen in the earlier observational studies, and were induced with both vaccines after one dose and increased after the second and third doses. Antibody geometric mean concentrations peaked at 1 month and then plateaued from month 7 for the single dose arm and peaked at month 7 then declined by month 24 for the two-dose and three-dose arms but stayed stable in the one-dose arms to 2 years. Single dose HPV 16 seropositivity at 24 months post dose was non-inferior to two and three doses and HPV 16 and HPV 18 avidity at month 24 did not differ by dose or vaccine. Both vaccines were well tolerated at all doses.

**Implications of all the available evidence**

A single dose of either the 2-valent or 9-valent HPV vaccine was both immunogenic and safe, with high rates of seroconversion and antibody levels stable to 2 years after vaccination and antibody kinetics similar to those seen in other settings where single-dose efficacy has been demonstrated. Higher antibody levels observed with the 2-valent vaccine compared to the 9-valent vaccine are consistent with earlier studies that also found both vaccines to be highly efficacious. A single dose of HPV vaccine would very significantly simplify vaccine delivery and reduce costs of implementing national HPV vaccination programmes, in turn potentially increasing vaccine introductions and uptake in the regions that urgently need cervical cancer prevention.

A single dose HPV vaccine would be simpler and cheaper to deliver than a multidose schedule but evidence is needed on the immunogenicity and efficacy of a single-dose schedule. Data from several observational studies in which some participants did not complete their allocated schedules suggest that a single dose of HPV vaccine provides efficacy against incident and persistent HPV 16 or 18 infection that is similar to efficacy with two or three doses.^6^ These include the IARC/India study of the 4-valent vaccine, Gardasil, and the Costa Rica Vaccine Trial (CVT) and PATRICIA trial that evaluated the 2-valent vaccine, Cervarix. In these studies, the frequency of 12-month persistent infection (a precursor for cervical cancer) with HPV 16 or HPV 18 was similar in females receiving a single dose compared with those receiving two or three doses. HPV 16 or HPV 18 IgG antibody seropositivity was high in all vaccinated groups, regardless of the number of doses received, but geometric mean concentrations (GMCs) were lower with one dose than with two or three doses. All HPV infection endpoints in these studies were significantly less frequent in participants receiving one dose compared with unvaccinated controls.^6^ Protection against persistent HPV16 or HPV18 infection after a single dose of the 2-valent vaccine was sustained up to 11 years in the CVT,^7^ and up to 9 years in the IARC/India study following a single dose of the 4-valent vaccine.^8^

The first randomised trial to examine the efficacy of a single dose of HPV vaccine in sexually active Kenyan women aged 15–20 years (KEN SHE) reported that, at 18 months post vaccination, the incidence of persistent HPV 16 or HPV 18 infection was 0·17/100 woman-years with both the 2-valent and the 9-valent vaccines, compared with 6·83 per 100 woman-years in the meningococcal vaccine control group.^9^ Vaccine efficacy for both HPV vaccines was 97·5%.

We report the results of the DoRIS trial in Tanzania, the first randomised trial to examine immune responses after a single dose of HPV vaccine in the target age group for HPV vaccination.

## Methods

### Study design

This open-label, randomised, phase 3, non-inferiority, immunobridging trial of two HPV vaccines was done in Mwanza, northwestern Tanzania (Dose Reduction Immunobridging and Safety Study of two HPV vaccines in Tanzanian girls [DoRIS]). The study was approved by the Tanzanian Medical Research Coordinating Committee and the ethics committee of the London School of Hygiene & Tropical Medicine. Regulatory approval was by the Tanzania Medicines and Medical Devices Authority.

### Participants

The trial protocol and procedures have been described previously.^10^ Briefly, we enrolled 930 girls aged 9–14 years living in Mwanza, Tanzania. Participants from 54 Government schools in Mwanza were invited to attend a research clinic in the city, after meetings with community leaders, school heads, teachers, and parents. Written informed consent was obtained from parents or guardians with written or fingerprinted assent from participants. Eligible participants were healthy (by medical history taken by clinician and physical examination if indicated) girls who were aged 9–14 years, HIV-negative following testing at screening, planning to reside in Mwanza for 36 months, and willing to give informed assent following informed consent from a parent or guardian. Exclusion criteria were previous HPV vaccination, history of cervical lesions or genital warts, past treatment for positive cervical cancer screening, pregnancy, being immunocompromised (including HIV infection), and being unwell based on medical history, clinical examination, or laboratory tests.

### Randomisation

Participants were randomly allocated (1:1:1:1:1:1) to one of six arms comprising three different dose schedules of two different HPV vaccines (three doses over 6 months, two doses given 6 months apart, or a single dose, for either the 2-valent vaccine [Cervarix, GSK Biologicals, Rixensart] or the 9-valent vaccine [Gardasil-9, Sanofi Pasteur MSD, Lyon]), using random permuted block sizes of 12, 18, and 24. An independent statistician computer-generated the randomisation list. Sequentially numbered sealed opaque envelopes concealed the allocation from the study team and participants. Once allocated, participants and clinic staff were unmasked. Participants were not masked as we did not think immune responses would be affected by girls knowing their vaccine group, and one of the trial's secondary aims was the acceptability of reduced-dose schedules.

### Procedures

We evaluated two prophylactic HPV virus-like particle (VLP) vaccines, both licensed by the US Food and Drug Administration and the European Medicines Agency. The 2-valent HPV vaccine (Cervarix; GSK Biologicals) is an HPV 16 and HPV 18 VLP vaccine containing L1 major capsid proteins of HPV 16 and HPV 18 and a proprietary adjuvant system (ASO4) that is formulated with monophosphoryl-lipid A adsorbed to aluminium hydroxide. The 9-valent vaccine (Gardasil-9; Sanofi Pasteur MSD) targets 9 genotypes (HPV 6, 11, 16, 18, 31, 33, 45, 52, and 58). The vaccine has an amorphous aluminium hydroxyphosphate sulfate adjuvant and each dose contains 60 μg of HPV 16 L1 protein and 40 μg of HPV 18 L1 protein. Both vaccines have excellent efficacy for preventing HPV 16 or HPV 18 associated grade 2 or grade 3 cervical intraepithelial neoplasia and HPV 16 or 18 associated adenocarcinoma in situ in women with no previous HPV 16 or 18 infection.^11^ There is no evidence of serious adverse events (SAEs) or adverse pregnancy outcomes with these vaccines.^2^, ^11^ The 2-valent vaccine demonstrates cross-protection to HPV 31, 33, and 45 infection and their sequelae.^11^ The 9-valent vaccine also prevents infection and high grade cervical, vaginal, and vulval disease associated with HPV 31, 33, 45, 52, and 58.^12^

At the screening visit, after informed consent, girls were screened for eligibility, including a medical history with clinical examination if warranted, HIV testing and counselling, and a urine pregnancy test. Girls were also asked to take a test of understanding (TOU) if aged 12 years or older to demonstrate appropriate understanding of the study. For girls younger than 12 years, a parent or guardian took the TOU. A screen failure was determined if the girl (or their parents) could not pass the TOU within three attempts.

Girls who had passed the screening process were invited to an enrolment visit within 30 days of the screening visit, at which eligibility was reconfirmed and girls were randomly allocated to one of the six arms. Blood samples were collected for immunogenicity assays and a dried blood spot was made for malaria testing by PCR. Girls were asked to provide two nurse-assisted, self-administered vaginal swabs for baseline HPV DNA testing and genotyping with the Anyplex II HPV 28 detection assay (Seegene, Seoul) done at the Catalan Institute of Oncology, Barcelona. Participants were then randomly assigned and vaccinated according to their study arm and were asked to attend the clinic 1 month after vaccination.

Subsequent vaccination visits were at 1 month after the first dose (for the second dose of the 2-valent vaccine three-dose arm) or at 2 months after the first dose (for the second dose of the 9-valent vaccine two-dose arm; appendix 2 p 9) and 6 months (girls enrolled in the two-dose and three-dose arms for either vaccine). At each vaccination visit, and at 6 months for the one-dose arms, we collected a dried blood spot for malaria testing. Participants were asked to attend the clinic 1 month after each vaccination visit for collection of information on adverse events (AEs). Whole blood samples of 15–20 mL (depending on participant's weight) were collected for immunological assays at 1, 7, 12 and 24 months after vaccination. Visit windows for vaccination and blood sampling visits were predefined in the protocol.^10^

All samples were processed at the Mwanza National Institute for Medical Research laboratory. HPV 16 and HPV 18 IgG concentrations were determined at the HPV Immunology Laboratory of the Frederick National Laboratory for Cancer Research in Maryland, USA, by use of an L1 VLP ELISA. This assay has previously been evaluated for monitoring antibody responses following single-dose HPV vaccination.^13^ Antibody seropositivity was defined as concentrations equal to or greater than the assay threshold (1·309 IU/mL for HPV 16 and 1·109 IU/mL for HPV 18). The HPV 16 and HPV 18 specific antibody avidity index in the ELISA was determined by the ratio of antibody concentrations in serum samples treated or not treated with Guanidine-HCl (GuHCl). Serum samples were tested at a dilution that yielded an absorbance reading of 1·0 (±0·5). GuHCl was added to the samples at various concentrations (0·5–3·5 M); the GuHCl concentration that reduced the optical density by 50%, compared with sample wells without GuHCl, defined the avidity index. HPV 16 and 18 specific memory B-cell responses and immune responses to the 7 other HPV genotypes in the 9-valent vaccine are being analysed separately and results are not included here.

### Outcomes

The primary outcome was non-inferiority of HPV 16 and HPV 18 specific seropositivity following one dose of HPV vaccine compared with two or three doses of the same vaccine 24 months after vaccination. This corresponded to two overall analyses: one evaluating the reduced dose schedule of the 2-valent vaccine, and one evaluating the 9-valent vaccine.

Vaccine immune responses were measured by the proportion of participants seroconverting to HPV 16 or 18, the GMC of HPV 16 and HPV 18 specific antibodies, HPV 16 and HPV 18 specific antibody avidity, and HPV 16 and HPV 18 specific memory B-cell responses.

Secondary objectives are evaluation of HPV 16 and HPV 18 seropositivity and antibody GMC after one dose versus two or three doses at other timepoints up to 24 months post-vaccination; comparison of HPV 16 and HPV 18 antibody responses after two versus three doses; and evaluation of HPV 16 and HPV 18 antibody avidity.

The trial had a coprimary immunobridging objective to demonstrate non-inferiority of HPV 16 and HPV 18 antibody GMC after one dose of vaccine compared with historical cohorts of women aged 10–25 years who received a single dose of HPV vaccine and in whom efficacy had been demonstrated; these results are reported in a companion publication.^14^

### Statistical analysis

With 155 participants in each arm, assuming a 20% loss-to-follow up over 36 months, we expected to have 130 girls in each arm 24 months after vaccination. If the true proportion seroconverting is the same in each arm, with 130 girls per arm, the study would have more than 90% power to demonstrate that the lower limit of the 95% CI for the difference (one-dose schedule–comparison schedule) is above –5%, indicating that seropositivity with the one-dose schedule was not decreased by more than 5·0%. This was the same non-inferiority margin that was used in the trials leading to licensure of the two-dose regimen in girls younger than 15 years.^15^, ^16^

Our power calculations were also based on our coprimary objective of demonstrating non-inferiority of GMCs in the immunobridging analyses. If the true GMC ratio (one-dose schedule:comparison schedule) between arms is 1·0, with 130 subjects in each arm, we would have more than 90% power to demonstrate that the lower limit of the 95% CI for the ratio of GMCs is above 0·50, indicating that the one-dose schedule does not decrease HPV 16 or HPV 18 antibody GMC by more than 50%. This non-inferiority margin was based on pre-established standards from the US Food and Drug Administration that were used in other HPV vaccine bridging trials.^15^, ^16^ We assumed an SD of 0·50–0·60 log_10_ anti-HPV concentration and used a one-sided non-inferiority test at the 2·5% level.

In non-inferiority trials, intention-to-treat (ITT) analyses can increase the risk of falsely claiming non-inferiority, since these analyses often lead to smaller observed effects than if all participants had adhered to the protocol.^17^ Therefore, the primary immunogenicity analyses were done in the per-protocol population, ie, participants who received the allocated doses of HPV vaccine in the protocol-defined window and who were HPV antibody negative and DNA negative at enrolment for the specific genotype (HPV 16 or HPV 18) under analysis. As a sensitivity analysis, we repeated all analyses in participants who received at least one dose of HPV vaccine (total vaccinated cohort), based on the arm to which they were randomised (ie, ITT). The total vaccinated cohort was used for the safety analysis. The analysis plan was finalised before the trial ended and was approved by the independent Data and Safety Monitoring Board.

Baseline characteristics were presented by arm. We tabulated the number and proportion of girls in each arm who were HPV 16 or 18 seropositive at each timepoint. For each vaccine type and HPV genotype, we calculated the difference (one-dose schedule–comparison schedule) in the proportion of girls who were seropositive, and estimated the 95% CI for the difference using the exact method of Chan and Zhang.^18^ Non-inferiority of seropositivity was concluded if the lower bound of the two-sided 95% CI for the difference was above –5%.

For each vaccine and HPV genotype, there were two primary hypothesis tests of non-inferiority of seropositivity 24 months after vaccination: one dose versus two doses, and one dose versus three doses (ie, a joint null hypothesis). Success was required for both tests to conclude non-inferiority; therefore, no adjustment for multiplicity was made to account for testing of multiple dose schedules. As a post-hoc sensitivity analysis, 97·5% CIs were calculated in accordance with the Bonferroni correction, to account for testing of multiple HPV genotypes.

For the analysis of antibody concentrations, we log_10_-transformed HPV genotype-specific antibody concentrations; those below the assay cutoff were given a value of half the cutoff before log transformation. The arithmetic mean log_10_ antibody concentration and 95% CIs for each arm were calculated, assuming a normal distribution. Log_10_ antibody concentrations were compared by use of a linear mixed effect model with log_10_ concentration as the response, dose group, timepoint, and a dose group time interaction term as fixed effects, and participant as a random effect to account for correlation of repeated measurements within participant. Separate models were used for each vaccine type and HPV genotype. The difference in log_10_ concentrations (reduced dose schedule–comparison schedule) and its 95% CI at each timepoint was estimated from the mixed effect model; the GMC ratio and 95% CIs were obtained by back-transformation. We used a similar analysis to compare antibody avidity between dose regimens, with separate models for each vaccine type and HPV genotype, and fixed effects for dose regimen, timepoint, and dose regimen–time interaction. For the secondary objectives, multiple comparisons were taken into account when interpreting the findings but no formal adjustments were made for multiplicity. This study was registered with ClinicalTrials.gov, NCT02834637.

### Role of the funding source

The funders of this study did not have any role in the study design, data collection and analysis, data interpretation, or writing of this report.

## Results

**Figure 1:**
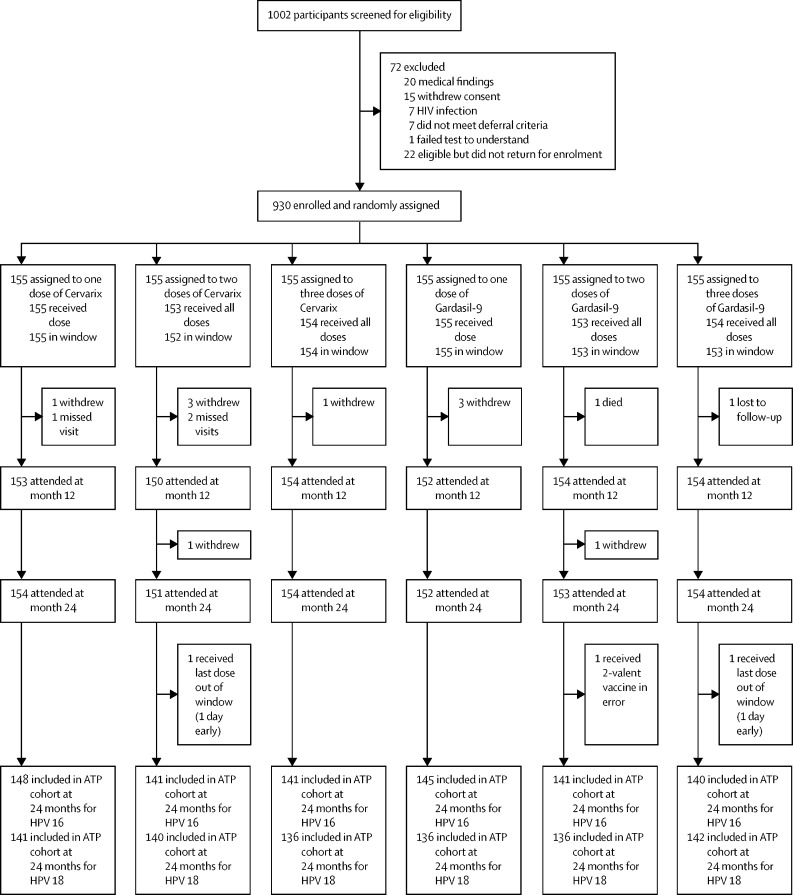
Trial profile ATP=according-to-protocol.

Between Feb 23, 2017, and Jan 6, 2018, we screened 1002 girls for eligibility. 50 girls were excluded at screening for medical findings (n=20), no consent (n=15), HIV infection (n=7), not meeting vaccine deferral criteria (n=7), or failing the TOU (n=1). 22 girls were eligible at screening but did not attend the enrolment visit. 930 (93%) of 1002 girls were enrolled and randomly assigned to receive one dose of Cervarix, two doses of Cervarix, three doses of Cervarix, one dose of Gardasil-9, two doses of Gardasil-9, or three doses of Gardasil (155 participants per group; figure 1). Of those enrolled, 922 (99%) received all scheduled doses within the protocol-defined window. Three girls withdrew, one was lost to follow-up and one died before completing her dose schedule. Two girls (one each in the two-dose 2-valent and three-dose 9-valent arms) received their 6-month dose one day early, and one girl in the two-dose 9-valent arm received the 2-valent vaccine in error. These eight girls were excluded from the per-protocol analyses but were included in the total vaccinated cohort analyses.

Baseline characteristics were similar between the six arms, with a median age of 10 years (IQR 9–12; table 1). 735 (79%) of 930 girls were in primary school, 555 (60%) lived with both parents, 117 (13%) had passed menarche, and 18 (2%) reported ever having had vaginal sex. Only 20 (2%) girls had evidence of any HPV infection on their vaginal swabs, of whom four were positive for HPV 16 or HPV 18 DNA. Overall, 57 girls (6%) were HPV 16 seropositive and 81 (9%) were HPV 18 seropositive at baseline.

**Table 1:** Patient demographics

		**1 dose 2-valent (n=155)**	**2 doses 2-valent (n=155)**	**3 doses 2-valent (n=155)**	**1 dose 9-valent (n=155)**	**2 doses 9-valent (n=155)**	**3 doses 9-valent (n=155)**	**Total (n=930)**
Age (years)		10 (9–12)	11 (10–12)	10 (9–12)	10 (9–12)	11 (10–13)	11 (9–13)	10 (9–12)
Age group								
	9–10 years	85 (54·8%)	74 (47·7%)	85 (54·8%)	88 (56·8%)	70 (45·2%)	73 (47·1%)	475 (51·1%)
	11–12 years	39 (25·2%)	45 (29·0%)	36 (23·2%)	41 (26·5%)	45 (29·0%)	41 (26·5%)	247 (26·6%)
	13–14 years	31 (20·0%)	36 (23·2%)	34 (21·9%)	26 (16·8%)	40 (25·8%)	41 (26·5%)	208 (22·4%)
Years lived in Mwanza								
	Entire life	116 (74·8%)	122 (78·7%)	121 (78·1%)	118 (76·1%)	121 (78·1%)	122 (78·7%)	720 (77·4%)
	>5 years	20 (12·9%)	18 (11·6%)	17 (11·0%)	18 (11·6%)	21 (13·5%)	14 (9·0 %)	108 (11·6%)
	≤5 years	19 (12·3%)	15 (9·7 %)	17 (11·0%)	19 (12·3%)	13 (8·4 %)	19 (12·3%)	102 (11·0%)
Living with								
	Mother	33 (21·3%)	32 (20·6%)	29 (18·7%)	31 (20·0%)	32 (20·6%)	39 (25·2%)	196 (21·1%)
	Father	6 (3·9 %)	5 (3·2 %)	4 (2·6 %)	6 (3·9 %)	6 (3·9 %)	2 (1·3 %)	29 (3·1 %)
	Both parents	93 (60·0%)	95 (61·3%)	97 (62·6%)	93 (60·0%)	86 (55·5%)	91 (58·7%)	555 (59·7%)
	Other	23 (14·8%)	23 (14·8%)	25 (16·1%)	25 (16·1%)	31 (20·0%)	23 (14·8%)	150 (16·1%)
Religion								
	Catholic	57 (36·8%)	59 (38·1%)	74 (47·7%)	73 (47·1%)	63 (40·6%)	67 (43·2%)	393 (42·3%)
	Other Christian	78 (50·3%)	77 (49·7%)	66 (42·6%)	68 (43·9%)	68 (43·9%)	73 (47·1%)	430 (46·2%)
	Muslim	20 (12·9%)	19 (12·3%)	15 (9·7 %)	14 (9·0 %)	24 (15·5%)	15 (9·7 %)	107 (11·5%)
School type								
	Primary	123 (79·4%)	122 (78·7%)	122 (78·7%)	127 (81·9%)	122 (78·7%)	119 (76·8%)	735 (79·0%)
	Secondary	32 (20·6%)	33 (21·3%)	33 (21·3%)	28 (18·1%)	33 (21·3%)	36 (23·2%)	195 (21·0%)
Passed menarche								
	Yes	20 (12·9%)	20 (12·9%)	19 (12·3%)	18 (11·6%)	20 (12·9%)	20 (12·9%)	117 (12·6%)
Ever cleansed vagina								
	Yes	15 (9·7 %)	15 (9·7 %)	13 (8·4 %)	14 (9·0 %)	12 (7·7 %)	19 (12·3%)	88 (9·5 %)
Ever had sex								
	Yes	1 (0·6 %)	2 (1·3 %)	5 (3·2 %)	1 (0·6 %)	4 (2·6 %)	5 (3·2 %)	18 (1·9 %)
Ever drank alcohol								
	Yes	0	0	0	0	0	0	0
	HPV 16 DNA positive	0	0	1 (0·6 %)	1 (0·6 %)	0	1 (0·6 %)	3 (0·3 %)
	HPV 18 DNA positive	0	0	2 (1·3 %)	1 (0·6 %)	0	0	3 (0·3 %)
Any high risk HPV genotype DNA								
	Yes	0	2 (1·3 %)	4 (2·6 %)	6 (3·9 %)	2 (1·3 %)	3 (1·9 %)	17 (1·8 %)
Any HPV genotype DNA								
	Yes	0	2 (1·3 %)	4 (2·6 %)	7 (4·5 %)	2 (1·3 %)	5 (3·2 %)	20 (2·2 %)
	HPV 16 seropositive	6 (3·9 %)	9 (5·8 %)	13 (8·4 %)	7 (4·5 %)	10 (6·5 %)	12 (7·7 %)	57 (6·1 %)
	HPV 18 seropositive	13 (8·4 %)	10 (6·5 %)	16 (10·3%)	16 (10·3%)	16 (10·3%)	10 (6·5 %)	81 (8·7 %)

Data are median (IQR) or n (%).

Retention at 24 months was 918 (99%) of 930 participants. At 24 months, we included 856 (93%) of the 918 girls attending in the per-protocol analysis of anti-HPV 16 antibody responses, and 831 (91%) in the per-protocol analysis of anti-HPV 18. All but two participants were seropositive for HPV 16 IgG antibodies at 24 months (one participant in each of the one-dose arms was not HPV 16 seropositive). All but six participants were HPV 18 seropositive at 24 months (two in the one-dose 2-valent vaccine group, three in the one-dose 9-valent vaccine group, and one in the three-dose 9-valent vaccine group were not HPV 18 seropositive). Non-inferiority of seroconversion of anti-HPV 16 antibodies at 24 months was met for one dose compared with two doses or three doses for both vaccines (table 2). Non-inferiority of HPV 16 seroconversion was also met when using a more stringent 97·5% CI, with the lower limit of the 97·5% CI of at least –4·6 for all comparisons (appendix 2 p 1). Although non-inferiority was not met for seroconversion for anti-HPV 18 antibodies, at least 98% of girls in the one-dose arms of both vaccines were anti-HPV 18 antibody positive at 24 months.

**Table 2:** Comparisons of antibody seropositivity after 1, 2 or 3 doses of human papillomavirus vaccine

		**1 dose**		**2 doses**		**3 doses**		Difference in seropositivity*(exact 95% CI)		
		n	Seropositive* (%)	n	Seropositive* (%)	n	Seropositive* (%)	1 dose–2 dose	1 dose–3 dose	2 dose–3 dose
**2-valent**										
HPV 16										
	Month 7	148	147 (99·3%)	142	142 (100·0%)	141	140 (99·3%)	−0·7% (−3·8 to 2·0)	0·0% (−3·1 to 3·4)	0·7% (−2·0 to 4·0)
	Month 12	147	146 (99·3%)	140	140 (100·0%)	141	141 (100·0%)	−0·7% (−3·8 to 2·0)	−0·7% (−3·8 to 2·0)	0†
	Month 24	148	147 (99·3%)	141	141 (100·0%)	141	141 (100·0%)	−0·7% (−3·8 to 2·0)	−0·7% (−3·8 to 2·1)	0†
HPV 18										
	Month 7	141	139 (98·6%)	141	141 (100·0%)	136	135 (99·3%)	−1·4% (−5·1 to 1·3)	−0·7% (−4·5 to 2·8)	0·7% (−2·0 to 4·1)
	Month 12	140	139 (99·3%)	139	139 (100·0%)	136	136 (100·0%)	−0·7% (−4·0 to 2·1)	−0·7% (−4·0 to 2·1)	0†
	Month 24	141	139 (98·6%)	140	140 (100·0%)	136	136 (100·0%)	−1·4% (−5·1 to 1·4)	−1·4% (−5·1 to 1·4)	0†
**9-valent**										
HPV 16										
	Month 7	144	144 (100·0%)	142	142 (100·0%)	140	140 (100·0%)	0†	0†	0†
	Month 12	145	145 (100·0%)	142	142 (100·0%)	140	140 (100·0%)	0†	0†	0†
	Month 24	145	144 (99·3%)	141	141 (100·0%)	140	140 (100·0%)	−0·7% (−3·9 to 2·0)	−0·7% (−3·8 to 2·1)	0†
HPV 18										
	Month 7	135	133 (98·5%)	137	137 (100·0%)	142	142 (100·0%)	−1·5% (−5·3 to 1·3)	−1·5% (−5·3 to 1·2)	0†
	Month 12	136	131 (96·3%)	137	137 (100·0%)	142	142 (100·0%)	−3·7% (−8·4 to −0·7)	−3·7% (−8·4 to −0·7)	0†
	Month 24	136	133 (97·8%)	136	136 (100·0%)	142	141 (99·3%)	−2·2% (−6·4 to 0·6)	−1·5% (−5·7 to 2·0)	0·7% (−2·1 to 4·0)

* Titres above the laboratory determined cutoff (HPV 16 1·309 IU/mL and HPV 18 1·109 IU/mL).

† Exact 95% CIs for the difference by Chan and Zhang^18^ method cannot be calculated because both proportions are 1·0, but there is still uncertainty around the point estimate.

Antibody GMCs at 24 months among girls in the per-protocol group who received one dose of the 2-valent vaccine were 23 IU/mL (95% CI 20–26) for HPV 16 and 10 IU/mL (95% CI 9–11) for HPV 18 (table 3). Among those receiving one dose of the 9-valent vaccine, GMCs were 14 IU/mL (95% CI 12–16) for HPV 16 and 6 IU/mL (95% CI 5–7) for HPV 18 at 24 months. As expected from previous studies, HPV 16 and HPV 18 antibody GMCs were higher among girls receiving two and three doses than among those receiving one dose (appendix 2 p 10) and were higher for HPV 16 than for HPV 18.^8^, ^10^ Among those receiving two doses of the 2-valent vaccine, HPV 16 antibody GMC was 163 IU/mL (95% CI 141–188) and HPV 18 antibody GMC was 50 IU/mL (95% CI 43–58) at 24 months. For those receiving two doses of the 9-valent vaccine, HPV 16 antibody GMC was 125 IU/mL (95% CI 107–146) and HPV 18 antibody GMC was 29 IU/mL (95% CI 25–35) at 24 months (table 3). For both HPV genotypes, antibody GMCs at 24 months were non-inferior when comparing two doses with three doses of the 9-valent vaccine (table 3). Antibody GMCs at 24 months among girls receiving three doses of the 2-valent vaccine were significantly higher than those receiving two doses (412 IU/mL *vs* 163 IU/mL for HPV16 and 107 IU/mL *vs* 50 IU/ml for HPV18, respectively), and non-inferiority was not met for either HPV genotype. Our immunogenicity results were similar among the total vaccinated cohort for both vaccines and both HPV genotypes (appendix 2 pp 2–3).

**Table 3:** HPV 16 and HPV 18 antibody GMCs at all visits, by dose group and vaccine in the per-protocol cohort

		**1 dose**		**2 doses**		**3 doses**		GMC ratio (95% CI)*2 doses or 3 doses
		N	GMC, IU/mL	N	GMC, IU/mL	N	GMC, IU/mL	
**2-valent**								
HPV 16								
	Day 0	149	<LLQ	145	<LLQ	141	<LLQ	..
	Month 1	149	48 (42–56)	144	52 (46–59)	141	50 (43–59)	..
	Month 7	148	16 (14–19)	142	1643 (1445–1868)	141	2658 (2221–3182)	0·62 (0·50–0·77)
	Month 12	147	19 (17–23)	140	268 (232–309)	141	641 (539–762)	0·42 (0·33–0·52)
	Month 24	148	23 (20–26)	141	163 (141–188)	141	412 (357–475)	0·39 (0·32–0·49)
HPV 18								
	Day 0	142	<LLQ	144	<LLQ	137	<LLQ	..
	Month 1	142	19 (16–22)	143	18 (15–21)	137	18 (16–21)	..
	Month 7	141	8 (6–9)	141	582 (505–670)	136	727 (607–870)	0·80 (0·64–1·00)
	Month 12	140	9 (7–10)	139	96 (83–111)	136	159 (132–190)	0·61 (0·48–0·76)
	Month 24	141	10 (9–11)	140	50 (43–58)	136	107 (90–126)	0·47 (0·38–0·59)
**9-valent**								
HPV 16								
	Day 0	148	<LLQ	143	<LLQ	141	<LLQ	..
	Month 1	147	55 (48–63)	143	51 (43–59)	141	57 (50–64)	..
	Month 7	144	16 (13–19)	142	1401 (1253–1566)	140	1025 (896–1174)	1·37 (1·11–1·68)
	Month 12	145	13 (12–15)	142	253 (219–291)	140	218 (189–251)	1·16 (0·95–1·42)
	Month 24	145	14 (12–16)	141	125 (107–146)	140	118 (102–137)	1·06 (0·87–1·30)
HPV 18								
	Day 0	139	<LLQ	138	<LLQ	143	<LLQ	..
	Month 1	138	20 (17–23)	138	17 (15–20)	143	19 (17–22)	..
	Month 7	135	7 (6–8)	137	400 (352–454)	142	383 (334–440)	1·04 (0·83–1·30)
	Month 12	136	5 (4–6)	137	59 (50–69)	142	67 (57–79)	0·87 (0·70–1·09)
	Month 24	136	6 (5–7)	136	29 (25–35)	142	32 (27–38)	0·91 (0·73–1·14)

Data are ELISA serum antibody GMC (95% CI) unless otherwise specified. LLQ=lower limit of quantitation. GMC=geometric mean concentration.

* Estimated with linear mixed effect model with log antibody titre as the response and dose group, timepoint, and a dose group-time interaction term as fixed effects, and participant as a random effect to account for correlation of repeated measurements within participant.

**Figure 2:**
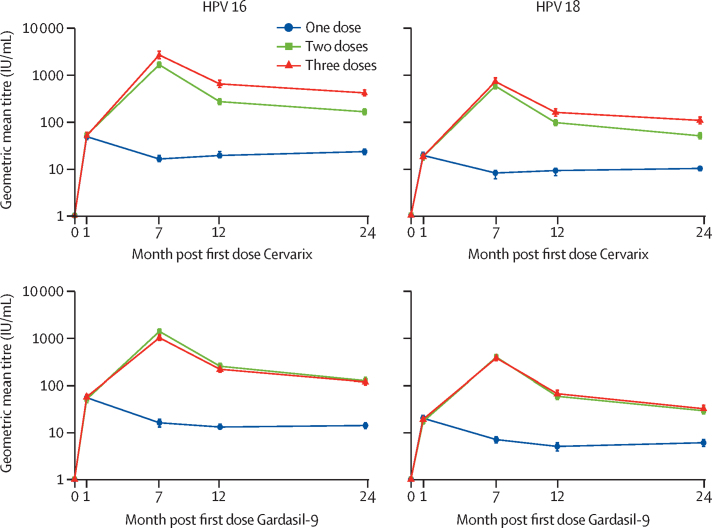
HPV 16 specific and HPV 18 specific antibody geometric means by number of HPV vaccine doses

Antibody GMCs among the two-dose and three-dose recipients peaked at 7 months and declined thereafter up until 24 months for both vaccines and both genotypes (figure 2). However, among one-dose recipients, HPV 16 and HPV 18 GMCs remained constant over time from 7 months to 24 months for both vaccines.

**Figure 3:**
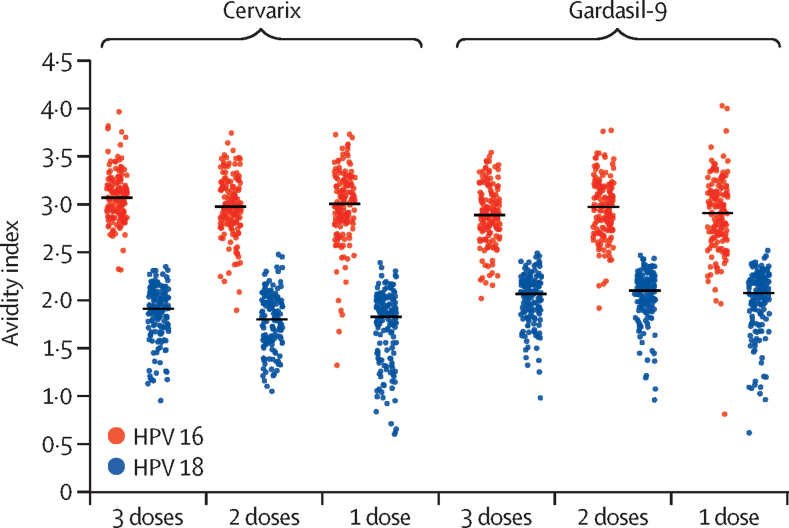
Distribution of HPV 16 and HPV 18 antibody avidity index at 24 months Each data point represents a single individual and the lines through the data points represents the median avidity index

By contrast with antibody GMCs, there was no evidence of a difference between the one-dose, two-dose schedules and three-dose schedules in GM antibody avidity index for HPV 16 or HPV 18 of either vaccine (appendix 2 p 5; figure 3). GM avidity index ratios were around 1·0 for all comparisons, with the lower limit of the 95% CI more than 0·90 in all but one comparison (GM avidity index ratio comparing one dose with three doses of the 2-valent vaccine 0·93, 95% CI 0·88–0·97).

53 SAEs were experienced by 42 (4·5%) of 930 girls by 24 months (appendix 2 pp 6–7). Clinical malaria hospital admission was the most common SAE (50 events, 39 girls). A 10-year-old girl in the two-dose 9-valent vaccine arm died from severe malaria 4 months after vaccination. There was no evidence of a difference between arms in the number of SAEs and no SAE was related to the vaccine. We recorded 573 non-serious AEs over 24 months with no evidence of a difference between arms. The most common events were skin conditions (n=128, 22% of events), gastrointestinal conditions (n=63, 11%), and helminth infections or amoebiasis (n=63, 11%; appendix 2 p 8).

## Discussion

This is the first randomised controlled trial to assess immune responses and safety of a single-dose HPV vaccine compared with two-dose or three-dose regimens among girls in the target age group for these vaccines. This is also the first trial in sub-Saharan Africa to examine immune responses to the currently recommended two-dose regimen compared with the originally recommended three-dose regimen. Our study is very timely since a randomised controlled trial in sexually active Kenyan women and girls aged 15–20 years (KEN SHE) has demonstrated excellent (>97%) efficacy with a single dose of either the 2-valent or the 9-valent vaccine against incident persistent HPV 16 or 18 infections at 18 months post-vaccination.^9^ Vaccine efficacy against a broader range of oncogenic genotypes (HPV16, 18, 31, 33, 45, 52, or 58) in that trial was 88·9%. Efficacy data from randomised trials are crucial for providing evidence to support recommendations for changes to a vaccine dose regimen. The data from our study in the target age for vaccination complement these results by demonstrating a high rate of seroconversion following a single dose of HPV vaccine and robust immune responses at 2 years post-vaccination. The WHO's Strategic Advisory Group of Experts recommended updating the HPV vaccine dose schedule to allow countries to choose a one-dose or two-dose schedule for girls aged 9–14 years and young women aged 15–20 years.^19^ Immunobridging of the DoRIS study results to KEN SHE immune responses is planned.

Consistent with previous studies, both vaccines in DoRIS were found to be well tolerated and no SAEs were considered related to vaccination. Malaria was the most common clinical event, which was not unexpected since malaria is endemic in the study area.

Our serology data support observations from non-randomised studies that a single dose of HPV VLP vaccines can induce strong and sustained IgG antibody responses up to 2 years post-vaccination. HPV 16 and HPV 18 antibody GMCs reached a plateau after 7 months that was sustained to 24 months. Ongoing follow-up of this cohort will allow us to determine if these antibody concentrations remain stable over time, as observed following one dose of the 4-valent vaccine in India, where antibody levels have been stable for 4 years and efficacy has been demonstrated for 9 years, and the 2-valent vaccine in Costa Rica, where antibody levels have been stable for 11 years and efficacy has been demonstrated for 11 years.^7^, ^8^ In our study, antibody responses to two and three doses of the vaccines peaked 1 month after the last dose and then declined thereafter to 24 months. The post-vaccination antibody kinetics we have observed provide reassurance that immune responses to these vaccines in sub-Saharan African girls are similar to those seen in other geographical regions.

As has been shown in the CVT, PATRICIA, and India/IARC studies, antibody GMCs following one dose of HPV vaccine in the DoRIS trial were lower compared with GMCs after two or three doses.^6^ However, these other studies have shown that protection provided by one dose against persistent HPV 16 and HPV 18 infection, the genotypes that cause 70% of cervical cancer cases, was no different than that offered by two and three doses.^6^, ^7^, ^8^ Encouragingly, the first randomised trial assessing the efficacy of one dose (KEN SHE) has demonstrated that a single dose of the 2-valent or 9-valent HPV vaccine had 97·5% efficacy against persistent HPV 16 and HPV 18 infection at 18 months compared with the control vaccine.^9^

There is no known immune correlate of protection for L1 VLP HPV vaccines, but antibody responses are considered essential in the protection conferred by these vaccines. A single dose of a VLP HPV vaccine might be sufficient to protect against HPV infection and its sequelae for several reasons.^20^ Passive transfer of serum or IgG from VLP-vaccinated animals to unvaccinated animals protects unvaccinated animals from papillomas associated with the cottontail rabbit papillomavirus.^21^ Antibodies induced by the virus neutralise the virus in vitro and, in addition, within-trial cross-protection and in vitro cross-neutralisation are also mirrored. The recombinant, type-specific L1 capsid proteins comprising the VLPs in the current HPV vaccines are highly immunogenic with a large number of repetitive epitopes that self-assemble and mimic HPV virions. ^22^ Similar arrays are known to induce long-lasting and stable humoral responses and it seems that the structure of VLP vaccines allow these vaccines to induce durable immune responses more characteristically seen with other viruses and live vaccines that present high density epitopes.^20^

Although we did not meet the non-inferiority criterion for anti-HPV 18 seropositivity at 24 months after a single dose of either vaccine, more than 98% of girls were anti-HPV 18 seropositive at that timepoint, and non-inferiority was met for anti-HPV 16 seropositivity. In the primary analyses, we did not adjust for multiplicity of testing. However, there remained good evidence of non-inferiority for HPV 16 when using a more stringent 97·5% CI in accordance with the Bonferroni correction.

The two-dose vaccine schedule is being offered in many countries, including in sub-Saharan Africa, following the change in recommendation from three to two doses.^23^ Several randomised trials previously reported non-inferior GMCs following two doses of the 2-valent and 4-valent HPV vaccines in young girls, compared with three-dose GMCs in women.^24^ In our study, where immune responses following two-dose and three-dose schedules were compared in the same age groups, HPV 16 and HPV 18 GMCs for two doses were non-inferior to three doses for the 9-valent vaccine, but non-inferiority was not met for the 2-valent vaccine where a third dose of the vaccine led to a further rise in antibody concentrations. Since an immune correlate of protection is undefined, the significance of this finding is unclear.

This is the first study in sub-Saharan Africa to measure HPV antibody avidity to any dose of HPV vaccine. Avidity is believed to reflect the degree of antibody affinity maturation and reflects how strongly the antibody binds to its target antigen. The India/IARC trial examined the HPV 16 antibody avidity index generated by the 4-valent vaccine in a subset of plasma samples by use of a modified HPV-L1 genotype-specific binding antibody assay.^25^ In that trial, the 18-month GM HPV 16 and HPV 18 antibody avidity index after one dose of the 4-valent vaccine was non-inferior to that after three doses. In the CVT, the avidity index increased with the number of doses of the 2-valent vaccine but, within each dose group, avidity index was stable up to 7 years.^26^ In the Netherlands, no difference was seen in HPV 16 antibody avidity at 5 years following one, two, or three doses of the 2-valent vaccine, but HPV 18 avidity was higher for one dose than for two or three doses.^27^ Some evidence suggests that antibody avidity might be affected by vaccine adjuvants. For hepatitis B vaccines, one study found that avidity maturation was more strongly promoted by the Adjuvant System (AS)01_B_, AS01_E_, AS03, and AS04 compared with the Alum adjuvant.^28^ However, although the 2-valent (containing the ASO4 adjuvant) and 9-valent vaccines have different adjuvants, we found no evidence of a difference between the different dose schedules in GM antibody avidity index for HPV 16 or HPV 18, and the GM antibody avidity index at 24 months was similar between the vaccines when given at the same dose, suggesting that other factors might influence the antibody affinity maturation for these vaccines.

Our study found that vaccination with the 2-valent vaccine resulted in higher concentrations of HPV 16 and HPV 18 IgG antibodies compared with the 9-valent vaccine at any dose. These results are similar to other studies that compared the 2-valent and 4-valent vaccines.^29^ Despite this, both vaccines have extremely high efficacy against persistent HPV 16 and HPV 18 infection and related sequelae, such as grade 2 or higher cervical intraepithelial neoplasia. A study showed that, with a cost-effectiveness threshold of per-capita gross decimal product, a 2-valent vaccine (with cross-protection to other genotypes) would avert 17·2 million cervical cancer cases and the 9-valent vaccine would avert 18·5 million cervical cancer cases in Gavi, the vaccine alliance-eligible countries.^30^ Costing data from our study and from the national HPV vaccination programme in Tanzania suggest that a one-dose schedule would be cost-saving and that delivery could be done at costs that would make HPV vaccination a very cost-effective intervention.^31^

Study limitations include the sample size that did not allow us to evaluate efficacy of single dose HPV vaccination in this population. However, as has been done for other HPV vaccination studies and following recommendations arising from a WHO–IARC workshop in 2013, we have immunobridged our results to cohorts in which efficacy of a one-dose schedule has been demonstrated, and results are presented in the companion paper.^14^

Our study has several strengths. We enrolled girls in Tanzania which, like many countries in sub-Saharan Africa, bears a high burden of cervical cancer and associated mortality. The representativeness of the trial setting and study population will allow the results to be generalisable to other parts of the continent. The study population is also in the target age group for vaccination, allowing us to evaluate antibody responses to one and two doses over time as girls pass through puberty. The study had excellent retention at 2 years and nearly all participants were vaccinated according to protocol. Immunogenicity analyses for HPV 16 and HPV 18 immune responses were done in a laboratory with significant expertise in HPV serology that has participated in numerous studies of single-dose HPV vaccine responses.^18^ The inclusion of HPV 16 and HPV 18 antibody avidity is novel and encouragingly showed that a single dose of vaccine had similar avidity compared with two or three doses of the same vaccine.

Our findings show that, in healthy African girls living in a malaria-endemic region, a single dose of the 2-valent or 9-valent HPV vaccines was well tolerated and resulted in high seropositivity rates and induced stable vaccine responses that persisted to 2 years. Antibody kinetics were similar to other studies in older females in other countries. Follow-up of the DoRIS cohort is continuing to provide data on durability and stability of single dose immune responses. New vaccine efficacy results for single dose in sexually active women are encouraging and efficacy data from observational studies are available up to 9–11 years. A single-dose regimen could encourage countries that have not yet included HPV vaccines in their national vaccination programmes to now introduce these vaccines. A single dose might also allow countries to do one-off activities to reach girls who missed HPV vaccination, including during the COVID-19 pandemic, and to focus on achieving high one-dose coverage rates which could in turn provide faster herd immunity to unvaccinated individuals. All these steps will contribute to the WHO cervical cancer elimination strategy targets for 2030.

## Data sharing

De-identified participant data presented in this manuscript can be made available after publication following written request to the London School of Hygiene & Tropical Medicine and the Mwanza Intervention Trials Unit, Tanzania. Requests must be accompanied by a defined analysis plan addressed to the corresponding author which will be reviewed by the Mwanza Intervention Trials Unit Data Sharing Committee and senior investigators at the London School of Hygiene & Tropical Medicine. Requesting researchers will be required to sign a Data Access Agreement if approval is given.

## Declaration of interests

DW-J reports a grant from GSK Biologicals in 2007 for a previous study on safety and immunogenicity of Cervarix in Tanzania unrelated to this submitted work. All other authors declare no competing interests.

## Supplementary Materials

KiSwahili translation of the abstract
