# Supplementary material for: Immunogenicity and safety of one-dose human papillomavirus vaccine compared with two or three doses in Tanzanian girls (DoRIS): an open-label, randomised, non-inferiority trial
Source: Lancet Glob Health. 2022 Sep 13;10(10):e1473–84. doi: 10.1016/S2214-109X(22)00309-6 (PMC9638030; doi:10.1016/S2214-109X(22)00309-6)
Supplement: KiSwahili translation of the abstract [file mmc1.pdf]

THE LANCET
Global Health

Supplementary appendix 2

This appendix formed part of the original submission and has been peer reviewed. We post it as supplied by the authors.

Supplement to: Watson-Jones D, Changalucha J, Whitworth H, et al. Immunogenicity and safety of one-dose human papillomavirus vaccine compared with two or three doses in Tanzanian girls (DoRIS): an open-label, randomised, non-inferiority trial. *Lancet Glob Health* 2022; **10:** e1473–84.

Kingamwili na usalama wa dozi moja ya chanjo ya virusi vya papilloma ikilinganishwa na dozi mbili au tatu kwa wasichana wa Tanzania (DoRIS): Utafiti wa wazi, unaotumia mbinu ya bahati nasibu na kutokuwa duni.

Utangulizi

Inakadiriwa asilimia 15 ya wasichana wenye umri wa miaka 9-14 duniani kote wamechanjwa dhidi ya virusi vya papilloma ya binadamu (VIPABI) kwa kutumia utaratibu uliopendekezwa wa dozi mbili au tatu. Utaratibu wa kutoa dozi moja ya chanjo ungekuwa rahisi na nafuu. Tunaripoti matokeo ya kinga ya mwili na usalama wa dozi tofauti za chanjo mbili za VIPABI kwa wasichana wa kitanzania.

Mbinu

Kwenye utafiti huu wa wazi, wa kutumia mbinu ya bahati nasibu, wa hatua ya tatu na kutokuwa duni, tulisajili wasichana wenye afya njema waliokuwa na umri wa miaka 9 -14 kutoka shule za serikali, Mwanza, Tanzania. Washiriki waliostahili kushiriki waliwekwa kwa njia ya bahati nasibu kupokea dozi moja, mbili au tatu ya ama chanjo ya valenti 2 (Cervarix, GSK Biologicals, Rixensart) au chanjo ya valenti 9(Gardasil-9, Sanofi Pasteur MSD, Lyon). Matokeo ya msingi ilikuwa ni kuwepo kwa kingamwili mahsusi kwa VIPABI 16 au 18 baada ya dozi moja ikilinganishwa na dozi mbili au tatu za chanjo hiyo hiyo ya VIPABI miezi 24 baada ya kuchanjwa. Usalama wa chanjo ulitathminiwa kwa madhara yalitajwa na mshiriki baada ya kuulizwa ndani ya siku 30 baada ya kila chanjo na madhara ya chanjo ambayo yalitajwa na mshiriki bila kuulizwa katika kipindi hadi miezi 24 baada ya kuchanjwa au mpaka hudhurio la mwisho. Matokeo ya msingi yalihusisha washiriki waliofuata protokali na usalama wa chanjo uliangaliwa kwa washiriki wote waliochanjwa. Utafiti umesajiliwa kwenye clinicaltrials.gov, NCT02834637

Matokeo

Kati ya Februari 23, 2017 na Januari 6, 2018, wasichana wapatao 1002 walifanyiwa tathmini kuona kama wanastahili kushiriki kwenye utafiti. Wasichana 72 hawakuwa na sifa za kushiriki na 22 walikuwa na sifa za kushiriki lakini hawakuhudhuria hudhurio la uandikishaji. Wasichana 930 waliandikishwa na kwa kutumia njia ya bahati nasibu walipangwa kupokea dozi moja ya Cervarix(washiriki 155), dozi mbili za Cervarix(washiriki 155) na dozi tatu za Cervarix(washiriki 155), dozi moja ya Gardasil-9(washiriki 155), dozi mbili za Gardasil-9(washiriki 155) au dozi tatu za Gardasil-9(washiriki 155). Washiriki 922 walipokea dozi zote kwa kufuata utaratibu uliopangwa na ndani ya kipindi maalum kilichowekwa (watatu walijitoa, mmoja alipotea kwenye ufuatiliaji, na mmoja alifariki kabla ya kumaliza; wawili walipokea dozi zao za miezi sita kabla ya kipindi maalumu kilichopangwa na mmoja alipokea chanjo isiyo ya valenti sahihi kimakosa; washiriki wote 930 waliingizwa kwenye kundi la washiriki waliochanjwa. Washiriki 918 (99%) katika ya 930 waliweza kuhudhuria hadi mwezi wa 24. Katika kundi la washiriki waliofanya mahudhurio yao kwa kufuata protokali(ATP), mpaka mwezi wa 24, asilimia 99 ya washiriki waliopokea dozi moja ya chanjo yoyote ya VIPABI walikuwa na kingamwili (IgG) mahususi dhidi ya VIPABI 16, ikilinganishwa na asilimia 100 ya washiriki waliopokea dozi mbili na asilimia 100 ya washiriki waliopokea dozi tatu. Hii iliweze kutimiza kigezo kilichowekwa awali ya kutokuwa duni. Kingamwili mahususi dhidi ya VIPABI18 katika mwezi wa 24 haikukidhi kigezo cha kutokuwa duni kwa dozi moja ikilinganishwa na dozi mbili au dozi tatu kwa chanjo zote, ingawa zaidi ya asilimia 98 ya wasichana kwenye makundi yote ya chanjo walikuwa na kingamwili dhidi ya VIPABI 18. Matukio 53 ya madhara makubwa (SAE) yalitokea kwa washiriki 42 (4.5%) kati ya 930, tukio lililotokea mara nyingi kuliko yote lilikuwa kulazwa hospitali kwa ajili ya malaria. Msichana mmoja alifariki kwa ajili ya malaria. Idadi ya matukio ilikuwa sawa kwa makundi yote ya chanjo na hakuna matukio ya madhara makubwa ambayo yalihusishwa na kuchanjwa.

Tafsiri

Dozi moja ya valenti 2 au valenti 9 ya chanjo ya VIPABI kwa wasichana wa miaka 9- 14 ilitoa mwitikio thabiti wa kinga ya mwili hadi miezi 24, ikionyesha kuwa utaratibu wa kutoa dozi pungufu unaweza kufaa katika kinga dhidi ya maambukiz ya VIPABI kwa wasichana walio kwenye kundi la umri uliolengwa kwa ajili ya kuchanjwa.

.
