# Supplementary material for: Immunogenicity and safety of one-dose human papillomavirus vaccine compared with two or three doses in Tanzanian girls (DoRIS): an open-label, randomised, non-inferiority trial
Source: Lancet Glob Health. 2022 Sep 13;10(10):e1473–84. doi: 10.1016/S2214-109X(22)00309-6 (PMC9638030; doi:10.1016/S2214-109X(22)00309-6)

THE LANCET
Global Health

Supplementary appendix 2

This appendix formed part of the original submission and has been peer reviewed. We post it as supplied by the authors.

Supplement to: Watson-Jones D, Changalucha J, Whitworth H, et al. Immunogenicity and safety of one-dose human papillomavirus vaccine compared with two or three doses in Tanzanian girls (DoRIS): an open-label, randomised, non-inferiority trial. *Lancet Glob Health* 2022; **10:** e1473–84.

Supplementary Table 1. Comparisons of antibody seropositivity after 1, 2 or 3 doses of HPV vaccine at M24, with 97.5% confidence intervals to account for multiple comparisons

	1 dose		2 doses		3 doses		Difference in seropositivity[1] (exact 97.5% CI)		
	N	Seropositive[1] (%)	N	Seropositive[1] (%)	N	Seropositive[1] (%)	1 dose – 2 dose	1 dose – 3 dose	2 dose – 3 dose
**According to protocol (ATP) cohort[2]**									
**2-valent**									
HPV16	148	147 (99.3%)	141	141 (100.0%)	141	141 (100.0%)	-0.7% (-4.5- 2.9)	-0.7% (-4.5- 2.9)	0[3]
HPV18	141	139 (98.6%)	140	140 (100.0%)	136	136 (100.0%)	-1.4% (-5.9- 2.1)	-1.4% (-5.9- 2.2)	0[3]
**9-valent**									
HPV16	145	144 (99.3%)	141	141 (100.0%)	140	140 (100.0%)	-0.7% (-4.6- 2.8)	-0.7% (-4.7- 2.9)	0[3]
HPV18	136	133 (97.8%)	136	136 (100.0%)	142	141 (99.3%)	-2.2% (-7.3- 1.4)	-1.5% (-6.7- 2.7)	-0.7% (-4.7- 3.0)
**Total vaccinated cohort (TVC)[4]**									
**2-valent**									
HPV16	154	153 (99.4%)	151	151 (100.0%)	154	154 (100.0%)	-0.6% (-4.3- 2.6)	-0.6% (-4.4- 2.7)	0[3]
HPV18	154	152 (98.7%)	151	151 (100.0%)	154	154 (100.0%)	-1.3% (-5.5- 2.0)	-1.3% (-5.4- 2.0)	0[3]
**9-valent**									
HPV16	152	151 (99.3%)	153	153 (100.0%)	154	154 (100.0%)	-0.7% (-4.4- 2.6)	-0.7% (-4.5- 2.6)	0[3]
HPV18	152	149 (98.0%)	153	153 (100.0%)	154	153 (99.4%)	-2.0% (-6.5- 1.3)	-1.3% (-6.0- 2.7)	-0.6% (-4.4- 2.6)

[1]Seropositivity defined as titres above the laboratory determined cut-off (HPV16 = 1.309 IU/mL; HPV18 = 1.109 IU/mL). [2]DoRIS participants who were ELISA antibody negative and DNA negative at baseline (pre-vaccination) for the HPV genotype under analysis. [3]Exact 95% confidence intervals for the difference using method of Chang and Zhang cannot be calculated because both proportions are 1.0, but there is still uncertainty around the point estimate. [4]DoRIS participants who received at least one dose of vaccine, analysed in their randomised arm irrespective of doses or vaccine received, or their HPV DNA or serostatus at baseline.

Supplementary Table 2. Comparisons of antibody seropositivity post HPV vaccination with 1, 2 or 3 doses of 2-valent or 9-valent (total vaccinated cohort[1])

	1 dose		2 doses		3 doses		Difference in seropositivity[2] (exact 95% CI)		
	N	Seropositive[2] (%)	N	Seropositive[2] (%)	N	Seropositive[2] (%)	1 dose – 2 dose	1 dose – 3 dose	2 dose – 3 dose
**2-valent**									
**HPV-16**									
Month 7	154	153 (99.4%)	152	152 (100.0%)	154	153 (99.4%)	-0.6% (-3.7- 1.9)	0.0% (-3.0- 3.0)	0.6% (-1.9- 3.7)
Month 12	153	152 (99.3%)	150	150 (100.0%)	154	154 (100.0%)	-0.7% (-3.7- 2.0)	-0.7% (-3.7- 1.9)	0[3]
Month 24	154	153 (99.4%)	151	151 (100.0%)	154	154 (100.0%)	-0.6% (-3.6- 1.9)	-0.6% (-3.7- 1.8)	0[3]
**HPV-18**									
Month 7	154	152 (98.7%)	152	152 (100.0%)	154	153 (99.4%)	-1.3% (-4.7- 1.2)	-0.6% (-4.1- 2.4)	0.6% (-1.9- 3.7)
Month 12	153	152 (99.3%)	150	150 (100.0%)	154	154 (100.0%)	-0.7% (-3.7- 2.0)	-0.7% (-3.7- 1.9)	0[3]
Month 24	154	152 (98.7%)	151	151 (100.0%)	154	154 (100.0%)	-1.3% (-4.7- 1.3)	-1.3% (-4.7- 1.2)	0[3]
**9-valent**									
**HPV-16**									
Month 7	151	151 (100.0%)	154	154 (100.0%)	154	154 (100.0%)	0[3]	0[3]	0[3]
Month 12	152	152 (100.0%)	154	154 (100.0%)	154	154 (100.0%)	0[3]	0[3]	0[3]
Month 24	152	151 (99.3%)	153	153 (100.0%)	154	154 (100.0%)	-0.7% (-3.7- 1.9)	-0.7% (-3.7- 1.9)	0[3]
**HPV-18**									
Month 7	151	149 (98.7%)	154	154 (100.0%)	154	154 (100.0%)	-1.3% (-4.7- 1.2)	-1.3% (-4.7- 1.2)	0[3]
Month 12	152	147 (96.7%)	154	154 (100.0%)	154	154 (100.0%)	-3.3% (-7.6- -0.6)	-3.3% (-7.6- -0.6)	0[3]
Month 24	152	149 (98.0%)	153	153 (100.0%)	154	153 (99.4%)	-2.0% (-5.7- 0.6)	-1.3% (-5.1- 1.8)	0.6% (-1.9- 3.6)

[1]DoRIS participants who received at least one dose of vaccine, analysed in their randomised arm irrespective of doses or vaccine received, or their HPV DNA or serostatus at baseline. [2]Titres above the laboratory determined cut-off (HPV16 = 1.309 IU/mL; HPV18 = 1.109 IU/mL). [3]Exact 95% confidence intervals for the difference using method of Chang and Zhang cannot be calculated because both proportions are 1.0, but there is still uncertainty around the point estimate.

Supplementary Table 3. HPV16 and HPV18 antibody geometric mean titres (GMT) at all visits, by dose group and vaccine (total vaccinated cohort[1])

	1 dose		2 doses		3 doses		GMT ratio (95% CI)[3] 2 dose/ 3 doses
	N[1]	GMT[2] (95% CI) (IU/mL)	N[1]	GMT[2] (95% CI) (IU/mL)	N[1]	GMT[2] (95% CI) (IU/mL)	
**2-valent**							
**HPV 16**							
Day 0	155	<LLQ[4]	155	<LLQ[4]	155	<LLQ[4]	–
Month 1	155	50 (43 -57)	154	52 (46 -59)	155	51 (44 -59)	–
Month 7	154	16 (14 -19)	152	1641 (1453 -1854)	154	2501 (2084 -3002)	0.66 (0.53 -0.81)
Month 12	153	19 (17 -23)	150	267 (233 -307)	154	623 (530 -733)	0.43 (0.35 -0.53)
Month 24	154	23 (20 -26)	151	163 (142 -187)	154	402 (351 -461)	0.40 (0.33 -0.50)
**HPV 18**							
Day 0	155	<LLQ[4]	155	<LLQ[4]	155	<LLQ[4]	–
Month 1	155	19 (16 -22)	154	18 (16 -21)	155	20 (17 -23)	–
Month 7	154	8 (6 -9)	152	593 (517 -679)	154	708 (594 -845)	0.84 (0.68 -1.04)
Month 12	153	8 (7 -10)	150	93 (80 -107)	154	158 (134 -187)	0.59 (0.47 -0.72)
Month 24	154	10 (8 -11)	151	51 (44 -58)	154	106 (91 -124)	0.48 (0.39 -0.59)
**9-valent**							
**HPV 16**							
Day 0	155	<LLQ[4]	155	<LLQ[4]	155	<LLQ[4]	–
Month 1	154	56 (48 -65)	155	51 (44 -59)	155	59 (53 -66)	–
Month 7	151	16 (14 -20)	154	1376 (1237 -1531)	154	1017 (897 -1154)	1.35 (1.11 -1.65)
Month 12	152	14 (12 -15)	154	249 (217 -285)	154	218 (191 -249)	1.14 (0.94 -1.39)
Month 24	152	14 (12 -16)	153	123 (106 -142)	154	117 (102 -135)	1.05 (0.87 -1.28)
**HPV 18**							
Day 0	155	<LLQ[4]	155	<LLQ[4]	155	<LLQ[4]	–
Month 1	154	20 (17 -23)	155	17 (15 -20)	155	20 (17 -22)	–
Month 7	151	7 (6 -8)	154	398 (354 -449)	154	386 (338 -440)	1.03 (0.84 -1.27)
Month 12	152	5 (5 -6)	154	58 (50 -68)	154	68 (58 -79)	0.86 (0.69 -1.06)

[1]DoRIS participants who received at least one dose of vaccine, irrespective of their HPV DNA or serostatus at baseline. [2]ELISA serum antibody geometric mean titre (GMT). [3]Estimated with linear mixed effect model with log antibody titre as the response and dose group, time point, and a dose group-time interaction term as fixed effects, and participant as a random effect to account for correlation of repeated measurements within participant. [4]Lower limit of quantitation.

Supplementary Table 4. Comparisons of geometric mean (GM) antibody avidity index (AI) after 1, 2 or 3 doses of HPV vaccine in DoRIS trial (ATP cohort[1])

	1 dose		2 doses		3 doses		Geometric mean AI ratio[3] (95% CI)		
	N	GM avidity index[2]	N	GM avidity index[2]	N	GM avidity index[2]	1 dose / 2 dose	1 dose / 3 dose	2 dose / 3 dose
**2-valent**									
**HPV-16**									
Month 12	147	2.73 (2.66 -2.81)	140	2.82 (2.77 -2.88)	141	2.96 (2.92 -3.01)	0.97 (0.94 -0.99)	0.92 (0.90 -0.95)	0.95 (0.93 -0.98)
Month 24	148	2.95 (2.89 -3.02)	141	2.97 (2.91 -3.02)	141	3.08 (3.04 -3.12)	1.00 (0.97 -1.02)	0.96 (0.93 -0.99)	0.96 (0.94 -0.99)
**HPV-18**									
Month 12	140	1.57 (1.51 -1.64)	139	1.73 (1.68 -1.78)	136	1.79 (1.73 -1.84)	0.91 (0.87 -0.96)	0.88 (0.84 -0.93)	0.97 (0.92 -1.02)
Month 24	141	1.69 (1.62 -1.76)	140	1.76 (1.70 -1.81)	136	1.82 (1.77 -1.88)	0.96 (0.92 -1.01)	0.93 (0.88 -0.97)	0.96 (0.92 -1.01)
**9-valent**									
**HPV-16**									
Month 12	145	2.59 (2.51 -2.67)	142	2.86 (2.81 -2.92)	140	2.74 (2.68 -2.79)	0.90 (0.88 -0.93)	0.95 (0.92 -0.98)	1.05 (1.01 -1.08)
Month 24	145	2.86 (2.79 -2.94)	141	2.94 (2.89 -3.00)	140	2.88 (2.82 -2.93)	0.97 (0.94 -1.00)	1.00 (0.96 -1.03)	1.02 (0.99 -1.06)
**HPV-18**									
Month 12	136	1.92 (1.86 -1.98)	137	2.03 (1.98 -2.08)	142	1.95 (1.90 -2.00)	0.95 (0.91 -0.98)	0.98 (0.95 -1.02)	1.04 (1.00 -1.08)
Month 24	136	1.98 (1.92 -2.05)	136	2.05 (2.01 -2.10)	142	1.99 (1.95 -2.04)	0.97 (0.93 -1.00)	1.00 (0.96 -1.03)	1.03 (0.99 -1.07)

[1]According to protocol: DoRIS participants who were ELISA antibody negative and DNA negative at baseline (pre-vaccination) for the HPV genotype under analysis. [2]Geometric mean avidity index. [3]Estimated with linear mixed effect model with log avidity index as the response and dose group, time point, and a dose group-time interaction term as fixed effects, and participant as a random effect to account for correlation of repeated measurements within participant.

Supplementary Table 5. Number of participants with at least one serious adverse event, and number of events, by trial arm from enrolment to Month 24 visit (total vaccinated cohort)

		1D 2-valent (N=155)	2D 2-valent (N=155)	3D 2-valent (N=155)	1D 9-valent (N=155)	2D 9-valent (N=155)	3D 9-valent (N=155)	Total (N=930)
**All SAEs**	Number of girls (%)	8 (5.2 %)	4 (2.6 %)	6 (3.9 %)	8 (5.2 %)	8 (5.2 %)	8 (5.2 %)	42 (4.5 %)
	(Number of events)	*(15)*	*(4)*	*(7)*	*(8)*	*(9)*	*(10)*	*(53)*
**Components of SAEs**								
Death	Number of girls (%)	0 (−)	0 (−)	0 (−)	0 (−)	1 (0.6 %)	0 (−)	1 (0.1 %)
	(Number of events)	*(0)*	*(0)*	*(0)*	*(0)*	*(1)*	*(0)*	*(1)*
Hospitalisation	Number of girls (%)	8 (5.2 %)	3 (1.9 %)	6 (3.9 %)	7 (4.5 %)	7 (4.5 %)	8 (5.2 %)	39 (4.2 %)
	(Number of events)	*(15)*	*(3)*	*(7)*	*(7)*	*(8)*	*(10)*	*(50)*
Life-threatening condition	Number of girls (%)	0 (−)	0 (−)	0 (−)	0 (−)	0 (−)	0 (−)	0 (−)
Persistent disability	Number of girls (%)	0 (−)	0 (−)	0 (−)	0 (−)	0 (−)	0 (−)	0 (−)
Congenital abnormality	Number of girls (%)	0 (–)	1 (0.6 %)	0 (–)	0 (–)	0 (–)	0 (–)	1 (0.1 %)
	(Number of events)	*(0)*	*(1)*	*(0)*	*(0)*	*(0)*	*(0)*	*(1)*
Other medically important event	Number of girls (%)	0 (–)	0 (–)	0 (–)	1 (0.6 %)	0 (–)	0 (–)	1 (0.1 %)
	(Number of events)	*(0)*	*(0)*	*(0)*	*(1)*	*(0)*	*(0)*	*(1)*

Supplementary Table 6. Serious adverse event by diagnosis and trial arm, from enrolment to Month 24 visit (total vaccinated cohort)

Number of events	1D 2-valent	2D 2-valent	3D 2-valent	1D 9-valent	2D 9-valent	3D 9-valent	Total
Severe malaria	14	3	3	6	9	9	44
Urinary tract infection	0	0	1	0	0	1	2
Gastroenteritis	0	0	0	1	0	0	1
Dehydration due to fever	1	0	0	0	0	0	1
Vasovagal syncope	0	0	2	0	0	0	2
Snake bite	0	0	1	0	0	0	1
Spontaneous abortion	0	0	0	1	0	0	1
Congenital anomaly	0	1	0	0	0	0	1
**Total events**	**15**	**4**	**7**	**8**	**9**	**10**	**53**

Supplementary Table 7. Number of non-serious adverse events[1], by trial arm, from enrolment to M24 visit (total vaccinated cohort)

Adverse event	1D 2-valent N events (% of all events)	2D 2-valent N events (% of all events)	3D 2-valent N events (% of all events)	1D 9-valent N events (% of all events)	2D 9-valent N events (% of all events)	3D 9-valent N events (% of all events)	Total N events (% of all events)
Malaria[2]	5 (6.5 %)	11 (10.9%)	5 (4.9 %)	2 (2.3 %)	7 (6.9 %)	13 (12.4%)	43 (7.5 %)
Fever / headache[3]	3 (3.9 %)	2 (2.0 %)	1 (1.0 %)	2 (2.3 %)	0 (–)	4 (3.8 %)	12 (2.1 %)
Skin / dermatological problem	21 (27.3%)	19 (18.8%)	25 (24.5%)	31 (35.6%)	12 (11.9%)	20 (19.0%)	128 (22.3%)
Gastrointestinal disorder[4]	5 (6.5 %)	8 (7.9 %)	9 (8.8 %)	12 (13.8%)	20 (19.8%)	9 (8.6 %)	63 (11.0%)
Respiratory disorder	7 (9.1 %)	9 (8.9 %)	8 (7.8 %)	3 (3.4 %)	13 (12.9%)	9 (8.6 %)	49 (8.6 %)
Urinary tract / renal disorder	5 (6.5 %)	5 (5.0 %)	8 (7.8 %)	3 (3.4 %)	3 (3.0 %)	7 (6.7 %)	31 (5.4 %)
Orthopaedic disorder	0 (–)	0 (–)	0 (–)	0 (–)	0 (–)	1 (1.0 %)	1 (0.2 %)
Helminth infection / amoebiasis /schistosomiasis	17 (22.1%)	9 (8.9 %)	10 (9.8 %)	10 (11.5%)	4 (4.0 %)	13 (12.4%)	63 (11.0%)
Accidental injury	3 (3.9 %)	4 (4.0 %)	5 (4.9 %)	0 (–)	1 (1.0 %)	2 (1.9 %)	15 (2.6 %)
Minor surgery/dental disorders	1 (1.3 %)	3 (3.0 %)	5 (4.9 %)	3 (3.4 %)	4 (4.0 %)	1 (1.0 %)	17 (3.0 %)
Eye disorder	2 (2.6 %)	7 (6.9 %)	9 (8.8 %)	5 (5.7 %)	18 (17.8%)	7 (6.7 %)	48 (8.4 %)
ENT disorder	2 (2.6 %)	10 (9.9 %)	8 (7.8 %)	7 (8.0 %)	7 (6.9 %)	11 (10.5%)	45 (7.9 %)
Haematological disorder	1 (1.3 %)	5 (5.0 %)	1 (1.0 %)	0 (–)	4 (4.0 %)	0 (–)	11 (1.9 %)
Sexually transmitted infection	0 (–)	0 (–)	1 (1.0 %)	0 (–)	0 (–)	1 (1.0 %)	2 (0.3 %)
Neurological disorder	2 (2.6 %)	3 (3.0 %)	2 (2.0 %)	2 (2.3 %)	2 (2.0 %)	0 (–)	11 (1.9 %)
Cardiovascular disorder	1 (1.3 %)	2 (2.0 %)	1 (1.0 %)	0 (–)	0 (–)	2 (1.9 %)	6 (1.0 %)
Gynaecological disorder	1 (1.3 %)	1 (1.0 %)	2 (2.0 %)	0 (–)	1 (1.0 %)	0 (–)	5 (0.9 %)
Musculoskeletal disorder	1 (1.3 %)	3 (3.0 %)	1 (1.0 %)	6 (6.9 %)	5 (5.0 %)	4 (3.8 %)	20 (3.5 %)
Other	0 (–)	0 (–)	1 (1.0 %)	1 (1.1 %)	0 (–)	1 (1.0 %)	3 (0.5 %)
**All non-serious AEs**	**77 (100%)**	**101 (100%)**	**102 (100%)**	**87 (100%)**	**101 (100%)**	**105 (100%)**	**573 (100%)**

[1]Note: more than one adverse event may be recorded for a participant on the same date, if symptoms are judged to be result of more than one condition. [2]Confirmed or suspected malaria. [3]Fever/headache without associated malaria. [4]Gastrointestinal disorder without associated malaria.

Supplementary Figure 1 Vaccination schedule by arm

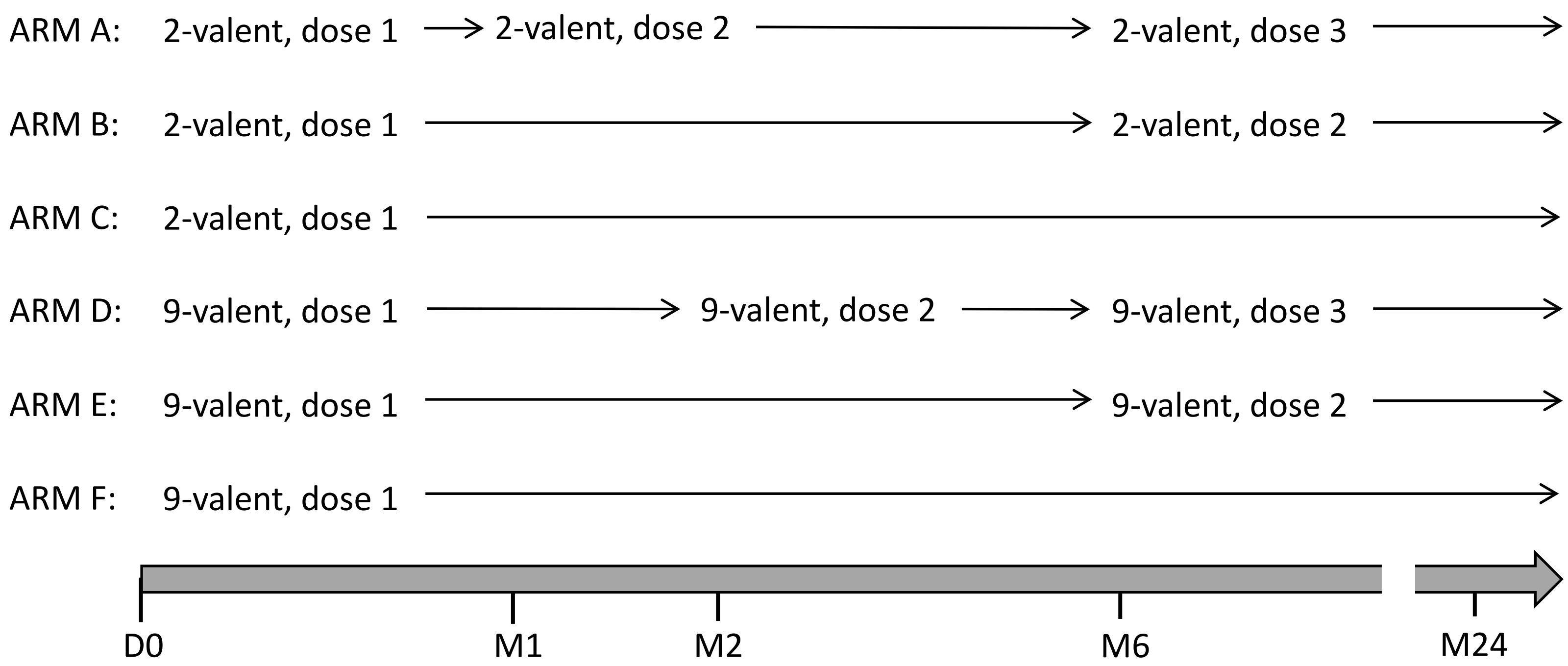

Supplementary Figure 2. Distribution of HPV-16 and HPV-18 antibody concentrations (IU/mL) at 24 months by arm. Each data point represents a single individual and the line through the data points represents the median concentration

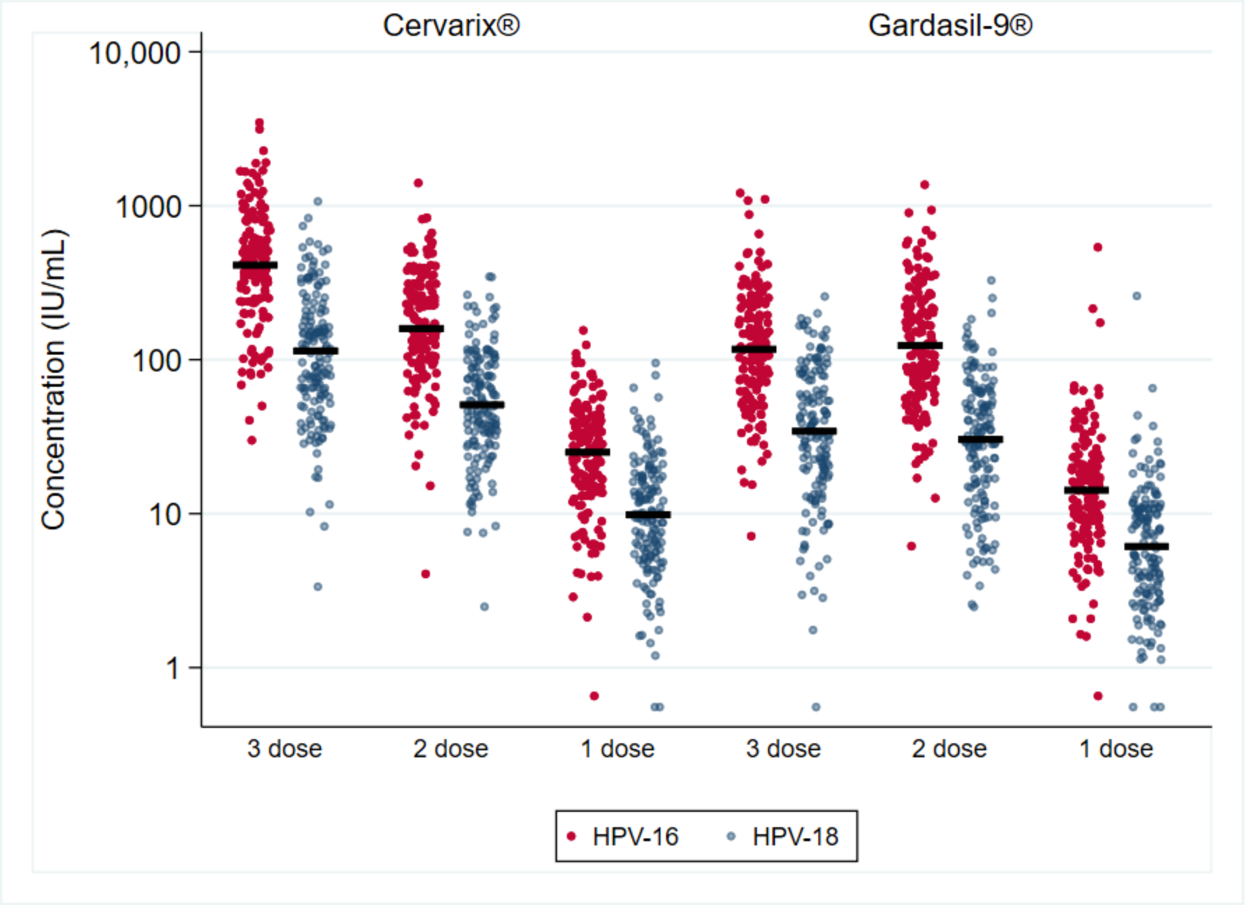

Supplement: Supplementary appendix 2 [file mmc2.pdf]
